# Extending risk-adapted care from radioiodine to surgery in differentiated thyroid carcinoma

**DOI:** 10.20945/2359-4292-2026-0110

**Published:** 2026-10-05

**Authors:** José Miguel Dora, Leonardo Barbi Walter, André B. Zanella, Iuri Goemann, Rafael Selbach Scheffel, Ana Luiza Maia

**Affiliations:** 1 Unidade de Tireoide, Hospital de Clínicas de Porto Alegre, Faculdade de Medicina, Universidade Federal do Rio Grande do Sul, Porto Alegre, RS, Brasil; 2 Divisão de Medicina Interna, Hospital de Clínicas de Porto Alegre, Porto Alegre, RS, Brasil; 3 Divisão de Endocrinologia, Hospital Nossa Senhora da Conceição, Grupo Hospitalar Conceição, Porto Alegre, RS, Brasil; 4 Departamento de Medicina Interna, Faculdade de Medicina, Pontifícia Universidade Católica, Porto Alegre, RS, Brasil; 5 Faculdade de Medicina, Universidade do Vale do Rio dos Sinos, São Leopoldo, RS, Brasil; 6 Departamento de Farmacologia, Instituto de Ciências Básicas da Saúde, Universidade Federal do Rio Grande do Sul, Porto Alegre, RS, Brasil

To the Editor,

We appreciate the thoughtful comments by Atallah and cols. on our nationwide analysis of radioiodine (RAI) prescription trends for differentiated thyroid carcinoma (DTC) in Brazil (^1^,^2^). Their proposal to extend risk-adapted, patient-centered principles from RAI prescription to the technical execution of therapeutic lateral neck dissection is a natural and welcome extension of the paradigm shift our data illustrate. It also resonates with the scientific literature and current guidelines/position statements (^3^), which similarly advocated risk-adapted decision-making across surgical extent, active surveillance, and adjuvant therapy.

As important as the technical aspects of surgery discussed in the letter by Atallah and colleagues is the number of thyroidectomies performed by year, particularly in the context of thyroid cancer. In Brazil, this is not solely a matter of individual surgeon experience, but also reflects a broader, structurally fragmented distribution of surgical volume nationwide. Our previous nationwide analysis identified 556 institutions performing thyroidectomy in Brazil in 2019. Nearly half (46.4%) performed fewer than 10 procedures annually, whereas only 5.2% performed >100 procedures/year, accounting for one-third of all operations (^4^). This landscape is worth bearing in mind alongside the proposal at hand: anatomical refinements that depend on careful preoperative imaging correlation, intraoperative judgment, and accumulated experience with compartment-oriented dissection will likely call for deliberate efforts to extend training to the many institutions that perform fewer than ten thyroidectomies per year.

The RAI de-escalation described in our study is supported by randomized clinical trials that directly compared different RAI activities or RAI omission against standard treatment, using long-term oncologic outcomes as endpoints. The surgical de-escalation with anatomical refinements proposed by the authors, selective fascia preservation, level IIb omission, and cutaneous nerve sparing, are so far supported mainly by retrospective, single-institution series, with heterogeneous conclusions: some cohorts found no difference in regional recurrence between selective and comprehensive dissection (8% versus 9% at five years) (^5^), while others reported a high prevalence of occult level IIa/IIb involvement favoring more complete clearance (^6^). We see this less as a limitation of the proposal than as an invitation to pursue the same prospective, outcome-focused research approach that has guided RAI de-escalation and is now being applied to thyroid cancer surgery, as exemplified by the ongoing ESTIMABL3 trial (NCT03570021), comparing prophylactic central neck dissection with no neck dissection.

In summary, we share the position that current guidelines define who should undergo lateral neck dissection more clearly than how it should be technically executed. Given the fragmentation of surgical volume shown in our national data, we would add that disseminating an anatomically refined technique would benefit from structured training and, ideally, a prospective, multicenter registry correlating technical choices with both oncologic outcomes and surgical morbidity across institutions of varying volume. Advancing risk-adapted, personalized care across every modality of DTC management, surgical and medical alike, remains a goal we are glad to pursue together.

## Data Availability

datasets related to this article will be avail-able upon request to the corresponding author.
